# Effectiveness of Leisure-Focused Occupational Therapy Interventions in Middle-Aged and Older People with Mild Cognitive Impairment: A Systematic Review

**DOI:** 10.3390/healthcare12242521

**Published:** 2024-12-13

**Authors:** Edgar Vásquez-Carrasco, Camila Huenchuquen, Catalina Ferrón, Jordan Hernandez-Martinez, Síbila Floriano Landim, Fabiola Helbig, Florencia Carmine, Pablo Valdés-Badilla, Cristian Sandoval, Celia Sánchez Gómez, Pedro Moruno-Miralles

**Affiliations:** 1 School of Occupational Therapy, Faculty of Psychology, University of Talca, Talca 3465548, Chile; edgar.vasquez@utalca.cl (E.V.-C.); camihuenchuquen@gmail.com (C.H.); catalina.ferron.f@gmail.com (C.F.); sibila.landim@utalca.cl (S.F.L.); fabiola.helbig@utalca.cl (F.H.); 2 Department of Physical Activity Sciences, Universidad de Los Lagos, Osorno 5290000, Chile; jordan.hernandez@ulagos.cl; 3 G-IDyAF Research Group, Department of Physical Activity Sciences, Universidad de Los Lagos, Osorno 5290000, Chile; 4 Programa de Investigación en Deporte, Sociedad y Buen Vivir, Universidad de los Lagos, Osorno 5290000, Chile; 5 Graduate Program in Health Promotion, Cesumar University (UniCesumar), Maringá 87050-900, Brazil; 6 Carrera de Medicina, Facultad de Medicina, Universidad de La Frontera, Temuco 4811230, Chile; f.carmine02@ufromail.cl; 7 Department of Physical Activity Sciences, Faculty of Education Sciences, Universidad Católica del Maule, Talca 3530000, Chile; 8 Sports Coach Career, School of Education, Universidad Viña del Mar, Viña del Mar 2520000, Chile; 9 Escuela de Tecnología Médica, Facultad de Salud, Universidad Santo Tomás, Los Carreras 753, Osorno 5310431, Chile; cristian.sandoval@ufrontera.cl; 10 Departamento de Medicina Interna, Facultad de Medicina, Universidad de La Frontera, Temuco 4811230, Chile; 11 Núcleo Científico y Tecnológico en Biorecursos (BIOREN), Universidad de La Frontera, Temuco 4811230, Chile; 12 Department of Developmental and Educational Psychology, University of Salamanca, 37008 Salamanca, Spain; 13 Department of Nursing, Physiotherapy and Occupational Therapy, University of Castilla-La Mancha, 45600 Toledo, Spain; pedro.moruno@uclm.es

**Keywords:** leisure activities, mild cognitive impairment, occupational therapy, people with MCI

## Abstract

**Background/Objectives:** This systematic review aimed to evaluate and synthesize scientific evidence on occupational therapy (OT) interventions focused on leisure activities to improve activities of daily living (ADLs) and cognitive function in middle-aged and older people with mild cognitive impairment (MCI). **Methods:** A systematic review was carried out following the guidelines established by the PRISMA statement. The study was registered in the PROSPERO database. Four databases were used for the literature search process (Scopus, Web of Science, Medline/PubMed, ScienceDirect), and selected results were assessed using standard tools for risk of bias and certainty of evidence with GRADEpro. **Results:** Of 169 records identified in the databases, 7 studies with a total of 620 middle-aged and older people (44.9% female) with a mean age of 77.5 years were analyzed using the PICOS format. The meta-analysis of the Mini-Mental State Examination (MMSE) revealed no significant improvements in cognitive function (*p* > 0.05). Individual studies reported varied results on ADL among people with MCI, with some demonstrating significant improvements following leisure interventions, while others found no notable differences between groups (*p* > 0.05). **Conclusions:** OT interventions did not significantly improve MMSE of the overall cognitive function and ADL performance in middle-aged and older people with MCI. Therefore, further studies detailing the dosage of interventions are needed.

## 1. Introduction

Aging becomes noticeable around age 30, with a more significant increase after age 40 [1]. By 2050, the population of individuals aged 60 or older is projected to reach 2 billion, accounting for approximately 22% of the world’s population [2]. One of the most prevalent neurological conditions among middle-aged and older people is mild cognitive impairment (MCI), which involves a moderate decline in cognitive performance across domains such as attention, executive function, learning, and memory [3]. According to the World Health Organization [4], MCI is characterized by a mild age-related decline in one or more cognitive domains, as well as other neuropsychological aspects, including memory, orientation, comprehension, judgment, language, and behavior. MCI affects approximately 15–20% of individuals aged 50 years and older worldwide, totaling nearly 55 million people [4]. Livingstone et al. [5] explain that MCI can begin as early as age 45 due to age-related brain changes and accumulated risk factors such as hypertension and diabetes.

A multidisciplinary approach to MCI care involves collaboration among various professionals to provide comprehensive support; this includes managing behavioral and psychological symptoms [6], facilitating access to community resources [7], and designing exercise programs that enhance physical fitness and cognitive function [8]. Research indicates that OT aids individuals with MCI in performing activities with greater independence and autonomy [9,10]. Addressing MCI from an OT perspective is essential for maintaining personal autonomy and occupational performance across all areas of life. Notably, individuals with MCI may experience a discrete decline in certain cognitive areas that do not significantly impact their ADLs or lead to social or work disabilities [11]. Previous studies have shown that therapeutic use of OT in individuals with MCI contributes to improvements in executive functions, occupational performance, interpersonal relationships, ADLs, and overall quality of life [12].

Different authors interpret leisure in various ways. While some authors define leisure as a fundamental occupation that implies a relationship between personal satisfaction and time dedicated to meaningful activities [13], other authors describe leisure as a fundamental part of the human experience that favors personal and social development [14]. In the same sense, Alban et al. [15] describe leisure as a fundamental right that contributes to a better quality of life and healthy lifestyles. In this sense, leisure activities are seen as a key tool for fostering holistic well-being and growth, both individually and collectively, particularly for middle-aged and older people with MCI as they promote engagement, cognitive stimulation, and social interaction through activities of interest [14,15].

Intervening in leisure activities through OT is critical because it has an impact on people with MCI’s quality of life and performance in ADL. Leisure is critical for maintaining independence and autonomy, as well as minimizing the progression of related diseases [16]. In everyday family and community contexts, leisure includes physical, recreational, and occupational activities and games [17]. Research suggests that engaging in cognitively demanding activities can postpone the onset of MCI and enhance overall quality of life [18]. Furthermore, participation in leisure activities acts as a buffer against the onset of diseases that affect cognition [19]. However, due to memory-related difficulties, people with MCI tend to reduce their participation in leisure activities [20]. Therefore, this systematic review with a meta-analysis aimed to evaluate and synthesize scientific evidence on OT interventions focused on leisure activities to improve the ADL and cognitive function in middle-aged and older people with MCI.

## 2. Methods

### 2.1. Protocol and Registration

This systematic review was conducted following the Cochrane Collaboration methodology [21]. Additionally, the report adhered to the PRISMA checklist and flowchart standards [22]. Additionally, the PROSPERO database registered the systematic review under the identification code CRD42023472129 [23].

### 2.2. Eligibility Criteria

The inclusion criteria for this systematic review were original studies peer-reviewed without language or publication date restriction, published until September 2024. Conference abstracts, books and book chapters, editorials, letters to the editor, records of protocol, reviews, case studies, and essays were excluded. In addition, the population, intervention, comparator, outcome, and study design (PICOS) framework was followed to incorporate the studies in a systematic review (Table 1).

### 2.3. Information and Database Search Process

Four databases were used for the literature search process; these were Scopus, Web of Science (core collection), Medline/PubMed, and ScienceDirect. They were used with US National Library of Medicine medical subject headings (MeSHs) and phrases in free language related to leisure, MCI, and OT. The following was the used search string (i.e., PubMed): ((((“Leisure Activities” [Mesh]) OR “Hobbies” [Mesh]) OR ((((((((((((((((Recreational Activities)) OR (Pastimes))) OR (Amusements)) OR (Leisure Pursuits)) OR (Relaxation Activities)) OR (Enjoyment Activities)) OR (Entertainment)) OR (Diversions)) OR (Unwinding Activities)) OR (Pleasure Activities)) OR (Rest Recreation)) OR (Playtime OR Spare-time Activities)) OR (Play Relaxation OR Free-time Activities)))) AND ((“Occupational Therapy” [Mesh]) OR ((Occupational therapy interventions) OR (Occupational therapist)))) AND (((“Cognitive Dysfunction” [Mesh]) OR “Neurocognitive Disorders” [Mesh]) OR ((((Cognitive Impairment Syndrome) OR (Early Cognitive Decline)) OR (Mild Cognitive Changes)) OR (Minor Cognitive Impairment))).

### 2.4. Study Selection and Data Collection Process

The studies were exported to Mendeley Reference Manager Version 2.116.1. Independent searches were conducted by three authors (CFF, CHB, and EVC), who also screened titles, abstracts, and full texts and removed duplicates. No disparities were discovered. Potentially acceptable studies were then re-examined in the full text and the rationale for removing those that did not meet the selection criteria was revealed. Finally, the entire data selection and extraction process was analyzed globally by two authors independently (CSG and PMM).

### 2.5. Methodological Quality Evaluation

The level of evidence and methodological quality of the studies were evaluated according to the criteria of the Oxford Center for Evidence-Based Medicine scale [24]. Level 1a studies were included (a systematic review of homogeneous randomized controlled trials (RCTs) with or without a meta-analysis). Studies with levels of evidence 1b, 2a, 2b, 3a, 3b, 4, and 5 were excluded. RCTs were downgraded if there were concerns about the risk of bias, consistency, accuracy, precision, transparency of results, or publication bias [24,25].

### 2.6. Data Collection Process

Relevant data were extracted from each study included in the systematic review and entered a data extraction form based on Cochrane recommendations [21], using Microsoft Excel^®^ software, version 16.89. The data extraction process was carried out by the authors (EVC, CFF, and CHB) independently, and then the results of each individual analysis were compared. Finally, the entire extraction process was jointly supervised by PMM and CSG. Data were extracted from each of the selected studies using the following variables: References, Oxford Level of Evidence, Participants, Intervention, Assessments, Data Collection Instruments, and Main Outcomes.

### 2.7. Risk of Bias Assessment

The review utilized the ROB2 method [21] to assess the risk of bias in the included randomized trials. Two authors (CFF and CHV) independently conducted the initial analysis, after which additional authors (EVC and PMM) reanalyzed the original studies and resolved any discrepancies through discussion, ultimately reaching a consensus.

### 2.8. Measures for Meta-Analysis

The study methodology includes a meta-analysis, with full information available on PROSPERO (registration code: CRD42023472129). The standardized mean difference (SMD), a statistic that assesses the absolute difference between mean values in two groups in a randomized controlled trial, was calculated for each analysis using Comprehensive Meta-analysis Software (RevMan 5.4), with a *p* value of <0.05 considered statistically significant [26]. In each trial, the random-effects model (Der Simonian–Laird approach) was employed to calculate and pool the SMD and mean difference (MD) of cognitive function from pre-intervention to post-intervention, comparing experimental and control groups [27]. The fundamental premise of the random-effects model is that true effects (e.g., interventions, duration) vary across studies, with samples drawn from populations with different effect sizes. If at least three studies yielded consistent results, the data were pooled [28]. Heterogeneity between trial results was tested using the Cochran Q test [29] and the I^2^ statistic. I^2^ values of <25%, 25–50%, and >50% indicate small, medium, and large inconsistencies, respectively [27]. Egger regression tests were also performed to detect small study effects and potential publication bias [30].

### 2.9. Certainty of Evidence

Studies were assessed for certainty of evidence using the Grading of Recommendations, Assessment, Development, and Evaluation (GRADEpro) scale [31]. They were classified as having high, moderate, low, or very low certainties of evidence. Due to the inclusion of studies with a randomized controlled trial design, all analyses started with a high degree of certainty. Concerns about the risk of bias, consistency, accuracy, precision, or transparency of results led to a downgrade. The studies underwent independent evaluation by two authors (CFF and CHB), with any discrepancies resolved by consensus with the other authors (EVC and PMM).

## 3. Results

**Figure 1:**
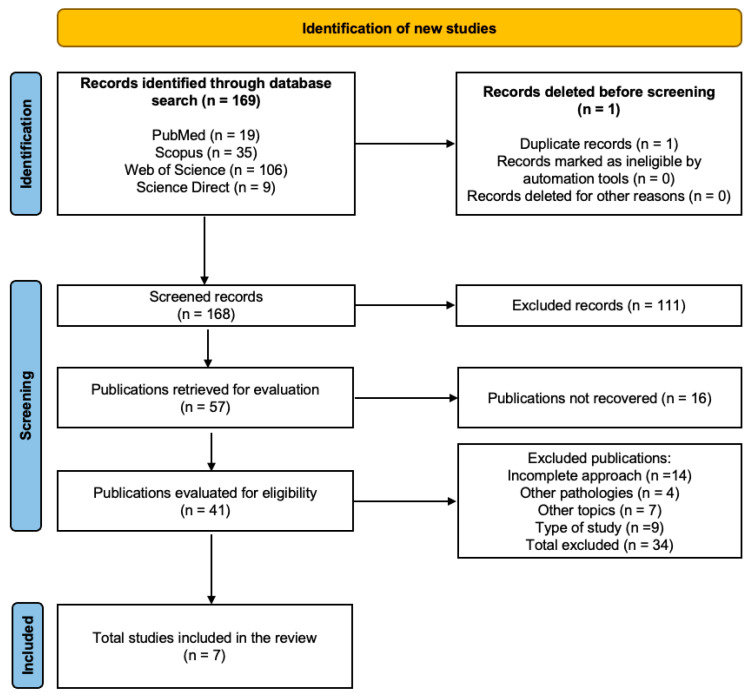
Flowchart of systematic review.

A total of 169 studies were identified across databases, with 41 studies undergoing full-text review. The flowchart specifies the reasons for excluding 133 studies that did not meet the established eligibility criteria. Finally, seven studies [32,33,34,35,36,37,38] were analyzed using the PICOS format. The described search results are presented using a flowchart established by the PRISMA statement, as illustrated in Figure 1 [39].

### 3.1. Methodological Quality

The quality of the evidence from the studies included in this systematic review with a meta-analysis is high because all seven studies are RCTs, reaching the highest level of evidence according to the Oxford Scale, specifically level 1a [32,33,34,35,36,37,38]. This design minimizes the risk of bias and provides a solid basis for reliably evaluating the impact of interventions.

### 3.2. Risk of Bias

**Figure 2:**
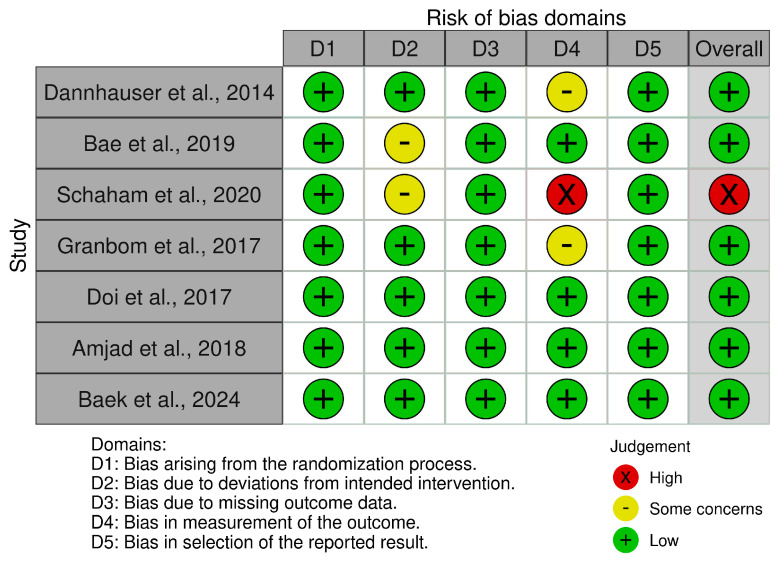
Risk of bias tools: traffic lights chart [32,33,34,35,36,37,38].

**Figure 3:**
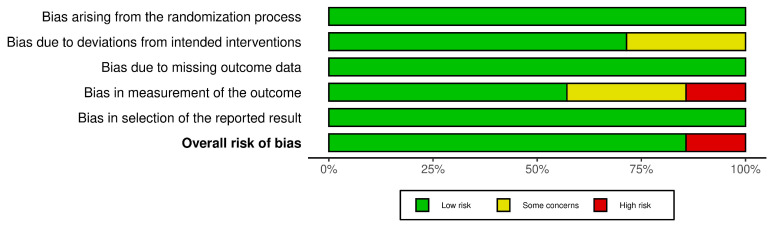
Risk of bias tools: Summary chart by domain.

Six studies had a low risk of bias [32,33,35,36,37,38] and one had high risk [34]. This suggests a low risk of bias in the research. Figure 2 and Figure 3 summarize the risk of bias.

### 3.3. Characteristics of the Studies

Of the seven studies reviewed, there was a positive impact of leisure activities on the quality of life perceived, cognitive functions, and performance of ADL of middle-aged and older people with MCI. Three studies concluded that participation in leisure activities for individuals with MCI can delay the onset of dementia and reduce the risk of developing MCI [32,33,34]. These findings underscore the importance of incorporating such activities into therapeutic interventions for people with MCI to enhance their cognitive resilience and overall well-being.

Based on the main outcomes or focus of the intervention, the seven reviewed studies were categorized into three groups: (i) leisure interventions that combined physical, cognitive, and social activities [32,33,36,38]; (ii) interventions that incorporated a single mode of leisure activities (dancing, music, reading newspapers or books, social activities, and playing cards or mahjong) [26,35]; and (iii) information and communication technology (ICT)-based interventions, which included tablet-based activities and specific cognitive stimulation applications [34,37]. The characteristics of each study and its outcome measures are described in Table 2.

### 3.4. Sample Characteristics

The total population of people with MCI obtained for the present systematic review was 620 middle-aged and older people (44.9% female), with a mean age of 77.5 years. The size of the representative sample and the number of participants in the studies varied from a minimum of 28 participants [34] to a maximum of 201 [36], considering the diversity of results and the type of program carried out.

### 3.5. Dosages and Interventions Performed

The studies incorporated leisure and physical activities as key components of OT interventions. Leisure activities, including recreational and cognitive tasks, aimed to enhance emotional well-being and social engagement [34,37]. Physical activities, structured to improve health, were also shown to delay cognitive decline [36,38]. These interventions promoted active participation, contributing to cognitive, emotional, and physical improvements across multiple studies [32,35,38].

The interventions varied in duration and frequency. Dannhauser et al. [32] applied 12 weeks of three weekly sessions for 30 min, while Bae et al. [33] used 24 weeks with two sessions per week for 90 min. Schaham et al. [34] and Granbom et al. [35] implemented 6 and 12 weeks, respectively, with one weekly session of 30 to 60 min. Doi et al. [36] carried out 40 weeks with one session per week of 60 min, and Amjad et al. [37] applied 6 weeks of five weekly sessions for 25 min. Baek et al. [38] followed 6 weeks with three weekly sessions for 40 min; all interventions had low to moderate intensity.

### 3.6. Activities of Daily Living

A meta-analysis was planned; however, it was not possible as only two studies assessed ADLs and in a heterogeneous manner. Two studies specifically assessed ADL outcomes using validated measurement tools; Baek et al. [38] reported significant improvements (*p* < 0.001) in ADL performance following a dual-task resistance exercise intervention. Granbom et al. [35], who assessed home-based interventions, found no significant differences (*p* = 0.601) in ADL outcomes between the experimental and control groups.

### 3.7. Cognitive Function

The reviewed studies examined the impact of leisure activities on cognitive function in middle-aged and older people with MCI. Three studies employed the MoCA [32,34,37]; the latter author does not report results from the control group, making it impossible to include the MoCA results in the meta-analysis. Individual results indicated that Amjad et al. [37] reported significant improvements in MoCA scores (*p* = 0.0001). Schaham et al. [34] also found improvements in MoCA scores in the experimental group using the TECH intervention, while the control group showed no significant improvements. Furthermore, Dannhauser et al. [32] observed cognitive improvements in their experimental group after the ThinkingFit Program, supporting the efficacy of structured cognitive interventions for individuals with MCI.

**Figure 4:**
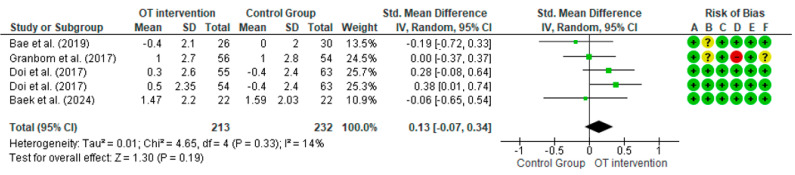
The effect of occupational therapy intervention compared to control groups on the following outcome: Mini-Mental State Examination. The squares indicate the study-specific effect estimate. Bars indicate the width of the corresponding 95% confidence interval. The diamond is centered on the summary effect estimate, and the width indicates the corresponding 95% confidence interval [33,35,36,38].

On the other hand, only the MMSE test could be meta-analyzed and it did not show significant improvements in favor of the TO intervention compared to the control groups (SMD = 0.13; 95% CI = −0.07 to 0.34; I^2^ = 14%; *p* = 0.19); these results are presented in Figure 4.

### 3.8. Certainty of Evidence

The results of the certainty of evidence did not allow us to make definitive recommendations on the use of OT interventions in leisure activities combined with cognitive stimulation (Table 3).

### 3.9. Adverse Effects and Adherence

The studies included in the systematic review did not report adverse effects and showed high adherence by participants, with few dropouts.

## 4. Discussion

### 4.1. Leisure Activities

The interventions reported in the studies highlight the potential benefits of incorporating physical and leisure activities into OT programs; leisure activities contributed to improved emotional well-being and social engagement, while physical activities played a key role in slowing cognitive decline [36,38]. Although cognitive stimulation from these activities appeared to enhance cognitive outcomes, the meta-analysis did not confirm significant improvements when evaluated through the MMSE, suggesting that the effectiveness of OT interventions remains inconclusive. The literature shows that participation in physical and cognitive activities by middle-aged and older people contributes significantly to maintaining cognitive functions and delaying the onset of dementia [40,41]. This could not be demonstrated in the meta-analysis of our systematic review. Furthermore, the combination of structured activities and leisure activities, as highlighted by Bae et al. [38], underscores the need for a holistic approach that improves not only physical health but also emotional and cognitive health, in line with the findings of neuroplasticity studies [42]. The studies by Doi et al. [36] and Baek et al. [38] show significant differences in the improvements observed between the experimental and control groups. Doi et al. [36] reported improvements in cognitive performance for the dance group (*p* = 0.026) and the music group (*p* = 0.008), indicating a moderate positive impact of these artistic interventions. In contrast, Baek et al. [38] found broader improvements in their experimental group, which included cognitive function, mood, depression, functional fitness, and ADL, all with *p* values less than 0.001. This suggests that Baek et al.’s [38] intervention was more effective in addressing multiple dimensions of participants’ health compared to the approach used by Doi et al. [36].

### 4.2. Activities of Daily Living

Individual results from the studies analyzed indicated variability in the impact on performance of ADLs. Across studies, Baek et al. [38] reported significant improvements following a dual-task exercise program lasting 6 weeks with three sessions per week for 40 min on ALDs (*p* < 0.001), whereas Granbom et al. [35] found no significant changes in ADL outcomes (*p* = 0.114) between groups following a traditional 12-week intervention. These discrepancies may be due to differences in intervention type and intensity. Baek et al. [38] used a dynamic dual-task approach, combining cognitive and physical tasks, while Granbom et al. [35] focused on traditional single-task activities, primarily targeting basic ADL training and social participation. Baek’s multitasking approach likely contributed to more significant cognitive and physical improvements. In the studies assessing ADLs, interventions varied in dosage; Baek et al. [38] implemented a more dynamic dual-task approach by integrating simultaneous cognitive and physical activities, such as walking while solving mental arithmetic problems or performing memory recall tasks. This dual-task method aimed to challenge both the participants’ physical coordination and cognitive processing, thereby enhancing their overall cognitive function and physical fitness [38]. In contrast, Granbom et al. [34] employed more standard interventions that focused on singular activities, such as traditional physical exercises or cognitive training sessions without combining the two elements simultaneously. This difference in approach likely contributed to the more significant improvements observed in the study of Baek et al. [38].

The improvement of ADLs in some studies suggests that dual-task activities, which integrate cognitive and physical components, might be effective in maintaining functional independence. This is consistent with studies showing that cognitive and physical multitasking can improve not only physical fitness but also executive function and task-switching ability, which are crucial for ADLs [43]. Furthermore, neurophysiological changes, including enhanced brain plasticity and improved connectivity between motor and cognitive regions, have been documented as a result of dual-task interventions, reinforcing their significance in cognitive and physical rehabilitation [44]. By fostering a synergistic relationship between cognitive and physical training, dual-task interventions may significantly enhance overall functional capacity and quality of life for older people, thereby contributing to sustained independence and well-being [45].

### 4.3. Cognitive Function

The meta-analysis of MMSE outcomes shows no significant improvements in cognitive function from OT interventions compared to control groups (*p* = 0.132). This suggests that while some studies reported improved MMSE scores [35,36,37], the overall evidence does not support consistent benefits across interventions. Cognitive assessments using the MMSE and MoCA reveal significant improvements (*p* < 0.05) in studies by Amjad et al. [37] and Baek et al. [38], which implemented interventions that combined cognitive stimulation with physical activity. For instance, Baek et al. [38] reported notable improvements in MMSE scores following a dual-task program lasting 6 weeks, with three sessions per week, each lasting 40 min/session, underscoring the positive impact of physical exercise on cognitive function (*p* < 0.001). Similarly, Amjad et al. [36] observed significant enhancements in cognitive performance (*p* = 0.004) with a 6-week intervention consisting of five sessions per week, each lasting 25 min/session, using cognitive games. However, despite these improvements in MMSE scores observed in multiple studies, Schaham et al. [34] and Granbom et al. [35] found no significant differences in cognitive outcomes between the intervention and control groups (*p* = 0.215). Granbom et al. [34] utilized an OT traditional 12-week intervention with one session per week lasting 60 min, while Schaham et al. [34] conducted a 6-week cognitive stimulation program with one session per week, each lasting 30 to 60 min, but reported no significant cognitive gains. This indicates that while physical and leisure activities contribute to cognitive preservation, the specific type of training, duration of interventions, and frequency of sessions may be critical to achieving consistent long-term cognitive improvements [37]. It highlights the need for longer follow-up periods to observe sustained cognitive benefits. Metzger et al. [46] highlight that interventions combining physical activity, music, and reminiscence therapy have been shown to be effective in improving cognition in older people with dementia and MCI. Reminiscence therapy had a significant impact on cognition (*p* < 0.05), while musical interventions, such as singing and music making, also showed significant improvements in cognitive performance (*p* < 0.01). These results suggest that integrating these strategies into OT may be key to improving individuals’ quality of life and emotional well-being.

### 4.4. Strengths and Limitations

Limitations of this systematic review include (i) the lack of statistically significant improvements in cognitive function from OT interventions based on MMSE results, limiting conclusions about their efficacy; (ii) variability in study designs, including differences in intervention types, intensity, and duration, complicating generalizability of findings; and (iii) inconsistency in impact on ADLs across studies, suggesting that factors such as baseline functional abilities and intervention types may significantly influence outcomes. Strengths include (i) the inclusion of diverse interventions highlighting the potential benefits of integrating physical, cognitive, and leisure activities into OT programs; (ii) a holistic approach that aligns with current research on aging and neuroplasticity; and (iii) emphasis on the need for longer follow-up periods to assess the sustainability of cognitive benefits, as current data do not adequately establish whether observed improvements in cognitive scores are maintained over time. Given the reported results, further studies are recommended to explore other aspects of cognitive function and establish protocols detailing the dosage (i.e., duration, volume, intensity, density) and types of activities performed. Such efforts will enhance the replication of successful interventions and optimize the design of evidence-based training programs aimed at improving cognitive function and independence in middle-aged and older people.

### 4.5. Practical Applications

This systematic review examines OT interventions that incorporate physical and leisure activities for middle-aged and older people with MCI. However, findings reveal inconsistencies in improvements related to ADLs and cognitive function, as evidenced by different outcomes across studies (e.g., [31,35,37]). These discrepancies underscore the need for more structured intervention protocols. Furthermore, it is essential to explore the role of technology in cognitive interventions, as illustrated by Schaham et al. [34]. Long-term follow-up studies are crucial to assess sustained effects on cognitive decline and ADL performance, reinforcing the need for culturally tailored OT programs given the increasing prevalence of MCI.

## 5. Conclusions

OT interventions did not significantly improve MMSE scores on general cognitive function and performance in ADLs in middle-aged and older people with MCI. However, individual studies’ results indicated that OT interventions incorporating physical and cognitive components may contribute to improved cognitive function and performance in ADLs. This suggests that while OT may have a positive impact on specific outcomes, the effectiveness of these interventions may depend on factors such as the type of activities included, the intensity of the intervention, and the frequency of sessions. Furthermore, the integration of dual-task exercises combining cognitive and physical tasks appears promising for improving both cognitive function and functional independence, supporting previous research on the benefits of neuroplasticity.

## Figures and Tables

**Table 1:** Selection criteria used in the systematic review with a meta-analysis.

Category	Inclusion	Exclusion
Population	Studies on a population with a mean age of 45 years or more and with MCI [2]. They were functionally independent and free of comorbid conditions or other debilitating social problems.	Studies with populations whose main pathology is different from MCI (chronic diseases, physical deterioration, or social problems) and under 45 years of age.
Intervention	Studies involving occupational therapy interventions or leisure-focused programs from 4 weeks onwards.	Studies whose main focus of intervention was not related to occupational therapy programs focused on leisure activities.
Comparison	Interventions with an experimental group focused on leisure activities and activities of daily living.	Lack of reference and/or follow-up data. Absence of control group.
Outcome	At least one assessment of cognitive functions and leisure and free time.	It does not present any assessment of these variables.
Study design	Experimental design studies (randomized controlled trial) with pre- and post-assessment.	Non-randomized, cross-sectional, retrospective, and prospective controlled studies.
Level of evidence	1a	1b, 2a, 2b, 3a, 3b, 4, and 5

MCI: Mild Cognitive Impairment.

**Table 2:** Selected studies of leisure activities and mild cognitive impairment.

Study	Country	Study Design	Sample’s Initial Health	Participants (*n*)	Mean Age (Years)	Type of Intervention and Control Groups	Training Volume			Training Intensity	ADLs (Assessment)	Cognitive Function (Assessment)	Main Outcomes	Effect Size
							Weeks	Frequency (Sessions/Week)	Session Duration (Minutes)					
[32]	Germany	RCT	Subjects diagnosed with MCI	EG: 32 CG: 35	73.9	ThinkingFit Program (EG) vs. Individual cognitive stimulation training (CG)	12	3	30	Low to moderate	Katz ADL Index	MMSE, MoCA	EG ↑ Significant improvements in physical fitness (*p* < 0.005). ↑ Improvement in cognitive outcomes after the intervention period. ↑ Significant improvements in quality of life (*p* < 0.05). CG ↔ Stable cognitive performance during the control period. ↔ No significant changes in quality of life during the control period.	Cognitive function, EG vs. CG, d: 0.465
[33]	Republic of Korea	RCT	Subjects diagnosed with MCI	EG: 41 CG: 42	75.5	Healthy activities in “Kenkojichi” (EG) vs. Health education (CG)	24	2	90	Moderate	not reported	TMT-A, TMT-B, MMSE	EG ↑ Cognitive activities associated with better overall cognitive performance (*p* = 0.678). ↔ Participation in social activities delays the onset of dementia and decreases the risk of MCI. ↑ Performing “favorite” activities plays a motivating and crucial role in the intervention. CG ↔ No significant changes in cognitive performance or MCI progression.	Cognitive function, EG vs. CG, d: 0.195
[34]	Israel	RCT	Subjects diagnosed with MCI	EG: 15 CG: 13	68.9	Cognitive stimulation program through TECH (EG) vs. Traditional cognitive training (CG)	6	1	30–60	Low to moderate	not reported	MoCA	EG ↑ TECH, a self-reported intervention, improves cognition and prevents MCI in adults. ↑ The intervention was engaging and motivating, aligned with participants’ interests. CG ↔ No significant improvements in cognition or MCI prevention without the intervention.	Cognitive function, EG vs. CG, d: 1.24
[35]	Sweden	RCT	Subjects diagnosed with MCI	EG: 80 CG: 73	81.4	Home-based interventions focused on social participation (EG) vs. Traditional home-based interventions (CG)	12	1	60	Low	Katz ADL Index	MMSE	EG ↔ No significant differences in social participation compared to CG (*p* = 0.114). ↔ No significant changes in important leisure activities (*p* = 0.601). CG ↔ Similar outcomes in social participation (*p* = 0.114). ↔ No significant differences in important leisure activities (*p* = 0.601).	Cognitive function, EG vs. CG, d: 0.17
[36]	Japan	RCT	Subjects diagnosed with MCI	EG: 134 CG: 67	76.2	Cognitive leisure activities (dance or music) (EG) vs. Health education (CG)	40	1	60	Low	not reported	TMT-A, TMT-B, MMSE	EG ↑ Greater improvement in MMSE scores compared to CG (dance: *p* = 0.026, music: *p* = 0.008). ↔ No significant differences in TMT-A and TMT-B scores compared to CG. CG ↔ No significant changes in MMSE scores. ↔ Similar outcomes in TMT-A and TMT-B scores.	Cognitive function, EG dance vs. CG, d: 0.080 Cognitive function, EG music vs. CG, d: 0.040
[37]	Pakistan	RCT	Subjects diagnosed with MCI	EG: 22 CG: 22	79.2	Xbox 360 Kinect cognitive games (EG) vs. Range of motion exercises (CG)	6	5	25	Low to moderate	not reported	MMSE, MoCA, TMT-A, TMT-B	EG ↑ Significant improvement in delta waves (0.673–0.029; *p* = 0.013). ↑ Significant improvement in theta waves (0.129–0.013; *p* = 0.002). ↑ Significant improvement in beta2 waves (0.044–0.009; *p* = 0.046). ↑ EEG complexity increased (0.051–0.042; *p* = 0.016). ↑ MMSE scores significantly improved (26.25–0.347 vs. 23.722–0.731; *p* = 0.003). ↑ MoCA scores significantly improved (25.65–0.310 vs. 22.00–0.504; *p* = 0.0001). ↑ TMT-A scores improved (1.429–0.234 vs. 2.225–0.259; *p* = 0.028). ↑ TMT-B scores improved (2.393–0.201 vs. 3.780–0.195; *p* = 0.0001). CG ↔ No significant changes in the above metrics.	Cognitive function, EG vs. CG, MMSE d: 2.528 MoCA d: 3.650 TMT-A d: 0.796 TMT-B d: 1.387
[38]	Republic of Korea	RCT	Subjects diagnosed with MCI	EG: 22 CG: 22	82.4	Dual-task resistance exercise (EG) vs. Resistance exercise (CG)	6	3	40	Low to moderate	Korean version of ADL	MMSE	EG (Intervention Group: Dual-Task Resistance Exercise and Resistance Exercise) ↑ Significant improvement in cognitive function (*p* < 0.001). ↑ Significant improvement in mood (*p* < 0.001). ↑ Significant improvement in depression (*p* < 0.001). ↑ Significant improvement in functional fitness (*p* < 0.001). ↑ Significant improvement in ADLs (*p* < 0.001). CG ↔ No significant changes compared to intervention groups.	Cognitive function, EG vs. CG, d: −0.02

ADLs: Activities of Daily Living; CG: Control Group; EG: Experimental Group; MCI: Mild Cognitive Impairment; MMSE: Mini-Mental State Examination; MoCA: Montreal Cognitive Assessment; TECH: Tablet Enhancement of Cognition and Health; TMT-A: Trail Making Test A; TMT-B: Trail Making Test B.

**Table 3:** Methodological Quality Assessment using GRADEpro tool.

Certainty of Evidence							Nº of Patients		Effect		Certainty	Importance
Nº of Studies	Study Design	Risk Assessment	Inconsistency	Indirect Evidence	Vagueness	Other Considerations	[Conventional Therapy plus Virtual Reality]	[Conventional Therapy]	Relative (95% CI)	Absolute (95% CI)		
**To analyze the effectiveness of the intervention focused on leisure as an occupation in people with MCI (follow-up: mean of 12 weeks; assessed with ANOVA)**												
1	RCT	It is not serious	It is not serious	It is not serious	It is not serious	None	63/63 (100%)	0/63 (0.0%)	Not estimable		++++ High	IMPORTANT
**To analyze the effectiveness of the intervention focused on leisure as an occupation in people with MCI (follow-up: mean of 24 weeks; assessed with TMT-A and TMT-B)**												
1	RCT	It is not serious	It is not serious	It is not serious	It is not serious	None	41/83 (49.4%)	42/83 (50%)	Not estimable		++++ High	IMPORTANT
**To analyze the effectiveness of the intervention focused on leisure as an occupation in people with MCI (follow-up: mean of six weeks)**												
1	RCT	Serious	It is not serious	It is not serious	It is not serious	None	28/28 (100%)	0/28 (0.0%)	Not estimable	See comments	+++ Moderate	IMPORTANT
**To analyze the effectiveness of the intervention focused on leisure as an occupation in people with MCI (follow-up: median of 12 years; assessed with Likert scale)**												
1	RCT	Serious	It is not serious	It is not serious	It is not serious	None	3544/3544 (100%)	0/3544 (0.0%)	Not grouped	See comments	+++ Moderate	IMPORTANT
**To analyze the effectiveness of the intervention focused on leisure as an occupation in people with MCI (follow-up: median of 1 year; assessed with multivariate regression model)**												
1	RCT	It is not serious	It is not serious	It is not serious	It is not serious	None	109/926 (11.8%)	817/926 (88.2%)	Not estimable		++++ High	IMPORTANT
**New outcome**												
1	RCT	It is not serious	It is not serious	It is not serious	It is not serious	None	134/201 (66.7%)	67/201 (33.3.%)	Not estimable	See comments	++++ High	IMPORTANT
**To analyze the effectiveness of the intervention focused on leisure as an occupation in people with MCI (follow-up: median of 24 weeks; assessed with trend analysis)**												
1	RCT	Serious	It is not serious	It is not serious	It is not serious	None	14/29 (48.3%)	14/29 (51.7%)	Not estimable		+++ Moderate	IMPORTANT

**Question:** [interventions focused on leisure] compared with [interventions not focused on leisure] for [persons with mild cognitive impairment]. CI: Confidence Interval; MCI: Mild Cognitive Impairment; TMT-A: Trail Making Test A; TMT-B: Trail Making Test B.
